# Developing a Simple Scoring System on CT Findings for Predicting Treatment Failure in *Mycobacterium avium* Complex Pulmonary Disease: The BCD (Bronchiectasis and Cavity Distribution) Score

**DOI:** 10.1093/ofid/ofaf565

**Published:** 2025-09-08

**Authors:** Makoto Hayashi, Hiroyasu Takishima, Hidekazu Cho, Fumihiro Yamaguchi, Takuya Yokoe, Satoshi Matsukura

**Affiliations:** Department of Respiratory Medicine, Showa Medical University Northern Yokohama Hospital, Yokohama-shi, Kanagawa-ken, Japan; Department of Respiratory Medicine, Showa Medical University Fujigaoka Hospital, Yokohama-shi, Kanagawa-ken, Japan; Department of Respiratory Medicine, Showa Medical University Northern Yokohama Hospital, Yokohama-shi, Kanagawa-ken, Japan; Department of Respiratory Medicine, Showa Medical University Fujigaoka Hospital, Yokohama-shi, Kanagawa-ken, Japan; Department of Respiratory Medicine, Showa Medical University Fujigaoka Hospital, Yokohama-shi, Kanagawa-ken, Japan; Department of Respiratory Medicine, Showa Medical University Fujigaoka Hospital, Yokohama-shi, Kanagawa-ken, Japan; Department of Respiratory Medicine, Showa Medical University Northern Yokohama Hospital, Yokohama-shi, Kanagawa-ken, Japan

**Keywords:** culture conversion, Mycobacterium avium-intracellulare infection, nontuberculous mycobacteria, outcome, response

## Abstract

**Background:**

Optimal timing for treatment initiation in *Mycobacterium avium* complex pulmonary disease (MAC-PD) remains unclear due to lack of established rules for predicting treatment response.

**Methods:**

A retrospective observational study was conducted to develop a prediction model for treatment failure at 2 Japanese university hospitals between 2012 and 2023. Participants were 135 patients with MAC-PD who received macrolides and ethambutol-containing regimens over 1 year. Treatment failure was defined as nonachievement culture conversion at 1 year. We selected model components as cavity (categorized by diameter) and bronchiectasis (categorized by modified Reiff score) on pretreatment computed tomography. Their combinations of each category were scored based on number of lobes involved and compared by average areas under the curve calculated using k-fold cross-validation.

**Results:**

Forty-three (31.9%) of the 135 patients failed in treatment. Number of lobes with cavities > 2cm or bronchiectasis with varicose or cystic changes was designated as the prediction model, with an average area under the curve of 0.798, and was named the Bronchiectasis and Cavity Distribution score. The representative metrics were sensitivity of 0.907 at the cutoff of 2 and specificity of 0.913 at the cutoff of 4 points. The patients were stratified into low-risk (0–1 points), intermediate-risk (2–3 points), and high-risk (4–6 points) groups. The treatment failure rates were 8.0%, 35.6%, and 69.2% in the respective groups.

**Conclusions:**

With simple assessment of computed tomography findings, the Bronchiectasis and Cavity Distribution score predicted treatment failure. Although validation studies are warranted, this score may provide guidance for treatment of MAC-PD.

## Teaser

The BCD score, number of lobes with cavities > 2 cm or varicose or cystic bronchiectasis on CT, predicted treatment failure in MAC-PD. This simple scoring system stratified patients by risk, which may provide a guidance for treatment of MAC-PD.

The incidence and prevalence of nontuberculous mycobacterial pulmonary disease (NTM-PD) are increasing worldwide, and the burden of the disease is concerning, with *Mycobacterium avium* complex (MAC) as the most common organisms [1–4]. MAC-PD is a potentially life-threatening disease, but several studies have reported that guideline-based treatment and culture conversion reduce its mortality [5–9]. These reports have highlighted the importance of timely initiation of treatment during the responsive phase in MAC-PD. However, the clinical course of MAC-PD varies from spontaneous culture conversion to death [10–13]. Thus, excessive treatment should be avoided because of the potential adverse effects associated with long-term multidrug treatment [5]. However, no clear guidance for determining the appropriate timing to treatment has been established. Several risk factors for treatment failure have been reported: male sex, older age, sputum acid-fast bacilli smear positivity, presence of cavity, and the BACES score (adding of body mass index, age, cavity, erythrocyte sedimentation rate, sex; originally a predictor for mortality) [14–17]. Nevertheless, these are insufficient for assessing treatment responsiveness in patients with MAC-PD because they are presented separately or in a scoring system including unalterable or nonspecific factors. As a result, determining when to initiate treatment is often challenging in MAC-PD. Being able to predict treatment response based on disease severity before treatment would facilitate the decision to initiate treatment. Therefore, we sought to develop a disease-specific and practical scoring system to predict treatment response in MAC-PD.

## METHODS

### Data Collection and Patient Selection

**Figure 1.**
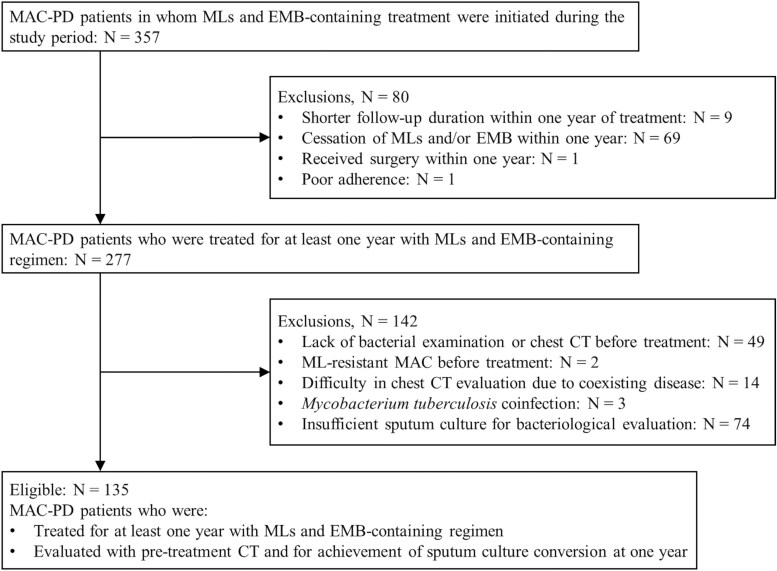
Inclusion and exclusion criteria of the present study. Abbreviations: CT = computed tomography; EMB = ethambutol; MAC-PD = *Mycobacterium avium* complex pulmonary disease; MLs = macrolides.

A retrospective observational study was conducted. We retrospectively reviewed electronic medical records of patients with MAC-PD who received treatment containing macrolides and ethambutol at Showa University Northern Yokohama Hospital between January 2012 and December 2023 and at Showa University Fujigaoka Hospital between January 2016, during which electronic medical records were introduced in the hospital, and December 2023. Both hospitals are located in Yokohama, Japan. We collected the patients' clinical data and chest computed tomography (CT) findings before treatment and bacteriological data longitudinally from before to 1 year of treatment initiation. The data were collected until January 2025. Diagnosis of MAC-PD was made according to the criteria in the official ATS/ERS/ESCMID/IDSA Clinical Practice Guideline [5]. Figure 1 shows the flowchart for inclusion and exclusion criteria in the present study. Exclusion criteria were as follows: shorter follow-up duration than 1 year of treatment, cessation of macrolides and/or ethambutol within 1 year from treatment initiation, addition of surgical treatment within 1 year from treatment initiation, poor adherence, lack of bacterial examination or chest CT before treatment, proven macrolide resistance of MAC before treatment, difficulty in evaluating the CT findings with MAC-PD due to coexisting pulmonary diseases, *M. tuberculosis* coinfection, and insufficient sputum culture for bacteriological evaluation. We evaluated treatment outcome through sputum culture conversion at 1 year from treatment initiation, which was defined according to the NTM-NET consensus statement [18] as the finding of at least 3 consecutive negative mycobacterial cultures from sputum collected at least 4 weeks apart. Treatment failure was defined as either failure to achieve culture conversion at 1 year after treatment initiation or MAC-PD-related death during the first year of treatment.

### Radiographic Evaluation

We evaluated chest CT findings performed within 6 months before treatment initiation. Radiographic characteristics were classified into 4 disease forms: noncavitary nodular-bronchiectatic form, cavitary nodular-bronchiectatic form, fibrocavitary form, and unclassifiable [16]. Bronchiectasis was evaluated using a modified Reiff score, which sums the degree of dilation in each lobe: cylindrical = 1, varicose = 2, and cystic = 3 (maximum score = 18) [19, 20]. The number of lobes with cavities of any size and the number of lobes with cavities > 2 cm in diameter were each counted. In counting number of lobes with bronchiectasis or cavities, the lingula was counted as a separate lobe from the left upper segment. Two pulmonologists who were blinded to the clinical and bacteriological data assessed these CT findings and made decisions through discussion in cases of disagreement.

### Statistical Analysis

#### Statistical Methods

Data are presented as numbers (percent) or median (interquartile range [IQR]). The chi-square test was used in comparisons of categorical variables or Fisher exact test was used instead when expected frequencies of any cell of contingency table were less than 5. The Wilcoxon rank-sum test was used in comparisons of ordinal and continuous variables. Multivariate logistic regression models were used for calculating odds ratios and developing receiver operating characteristic curves and area under curve (AUC) values. K-fold cross-validation (k = 5) was used to prevent overfitting. Interrater reliability was evaluated using weighted kappa. In all analyses, we considered *P* < .05 as significant. All data were analyzed using JMP Pro v17.0.0 (SAS Institute Inc, Cary, NC, USA), except for weighted kappa, which was calculated using R v 4.5 (R Foundation).

#### Development of a Predictive Model for Treatment Failure

We constructed a predictive scoring model for treatment failure at 1 year of treatment of MAC-PD according to previously published guidance [21]. The procedure follows the TRIPOD + AI statement [22]. To provide disease specificity for the scoring model, cavity and bronchiectasis were selected as candidate predictors based on previous studies [14–16, 23]. They were classified by each severity: cavities with any diameter and cavities > 2 cm in diameter, and all bronchiectasis, varicose or cystic bronchiectasis, and cystic bronchiectasis. The extent of these findings was expressed by numbers of involved lobes. Each of their effects on treatment failure were evaluated as odds ratios adjusted by other predictive factors previously reported [14–17]: age, sex, and sputum acid-fast bacilli smear. We created combinations to assess disease extension with cavities and bronchiectasis of each severity as the candidates for the scoring model. These candidates were constructed by combinations of the number of lobes with cavities and bronchiectasis, respectively, with each severity. They were calculated by the following 3 approaches: (1) summing the number of lobes with cavities of each severity and that with bronchiectasis of each severity (maximum 12 points); (2) counting the total number of lobes with either finding (maximum 6 points); and (3) counting the number with each finding independently (maximum 6 points). The predictive accuracies of these candidates were compared by average AUCs provided by k-fold cross-validation. The predictive model was designated from those candidates based on their predictive accuracy and clinical usability. We examined the predictive performance of the designated model and classified the study patients according to the risk of treatment failure. The classification was compared to the BACES scoring system for predictive accuracy in treatment failure. Patients with missing variables for the BACES score were excluded in this comparison.

### Ethical Approval Statement

The study protocol was conducted in accordance with the Declaration of Helsinki and approved by the Ethical Committee of Showa University, Tokyo, Japan (approval no.: 2023-167-B) with waiver of the requirement for informed consent because of the retrospective study design.

## RESULTS

### Treatment Response and Patient Characteristics

In total, 135 patients with MAC-PD were eligible for analysis (Table 1). Of these, 92 patients (68.1%) achieved culture conversion after 1 year of treatment, and 43 patients (31.9%) experienced treatment failure (42 patients failed to achieve culture conversion, and 1 patient died due to MAC-PD). Notably, the treatment failure group exhibited greater severity of both cavitary lesions and bronchiectasis than the culture conversion group: the frequency of patients with cavities > 2 cm was 21.7% in the culture conversion group and 44.2% in the treatment failure group, *P* = .007; and modified Reiff scores were 5 (IQR 3–8) and 9 (IQR 7–11), respectively, *P* < .001. The treatment failure group also showed lower serum albumin (4.1 [IQR 3.8–4.3] and 3.9 [IQR 3.4–4.1] g/dL, respectively, *P* < .001), higher C-reactive protein (0.10 [IQR 0–.36] and 0.32 [IQR .06–2.36] g/dL, respectively, *P* = .025), and frequent previous treatment for NTM-PD (15.2% and 32.6%, respectively, *P* = .024). The most frequently used treatment regimen was the combination of macrolides, ethambutol, and rifamycins in 121 (89.6%) of the 135 patients, followed by the combination of macrolides and ethambutol in 8 (5.9%) patients. Parenteral aminoglycosides were administered to 10 (7.4%) patients within 1 year of treatment initiation. Amikacin liposome inhalation suspension was not administered to any patient. No significant differences were shown for initial treatment regimen.

**Table 1.** Characteristics of the Study Patients With MAC-PD Divided into Culture Conversion Group and Treatment Failure Group

	Total	Culture Conversion	Treatment Failure	*P* Value
	N = 135	N = 92	N = 43	
Age (y)	71 (63–77)	71 (61–75)	73 (66–78)	.176
Women	72 (63–77)	71 (77.2)	31 (72.1)	.522
Etiological organisms				.075
*Mycobacterium avium*	111 (82.2)	71 (77.2)	40 (93.0)	…
*Mycobacterium intracellulare*	22 (16.2)	19 (20.7)	3 (7.0)	…
*Mycobacterium avium* and *Mycobacterium intracellulare*	2 (1.5)	2 (2.2)	0	…
Positive AFB smear	85 (63.0)	56 (60.9)	29 (67.4)	.461
Disease form				.208
Noncavitary NB	67 (49.6)	50 (54.4)	17 (39.5)	…
Cavitary NB	60 (44.4)	36 (39.1)	24 (55.8)	…
Fibrocavitary	6 (4.4)	5 (5.4)	1 (2.3)	…
Unclassifiable	2 (1.5)	1 (1.1)	1 (2.3)	…
Evaluation in cavitary lesion				
Patients with cavities	67 (49.6)	42 (45.7)	25 (58.1)	.176
No. of lobes with cavities	0 (0–1)	0 (0–1)	1 (0–2)	.026
Patients with cavities > 2 cm	39 (28.9)	20 (21.7)	19 (44.2)	.007
No. of lobes with cavities > 2 cm	0 (0–1)	0 (0–0)	0 (0–1)	.002
Evaluation in BE				
Modified Reiff score	6 (4–9)	5 (3–8)	9 (7–11)	< .001
Degrees of most severe BE				< .001
Cylindrical	21 (15.6)	19 (20.7)	2 (4.7)	…
Varicose	65 (48.1)	52 (56.5)	13 (30.2)	…
Cystic	48 (35.6)	20 (21.7)	28 (65.1)	…
No. of lobes with any BE	4 (3–5)	3 (2–5)	5 (4–6)	< .001
No. of lobes with varicose or cystic BE	2 (1–3)	1 (1–2)	3 (2–4)	< .001
No. of lobes with cystic BE	0 (0–1)	0 (0–0)	1 (0–2)	< .001
PPFE-like findings	44 (32.6)	27 (29.4)	17 (39.53)	.239
BACES score classification	N = 82	N = 59	N = 23	.055
Mild (0 or 1 point)	21 (25.6)	18 (30.5)	3 (13.0)	…
Moderate (2 or 3 points)	45 (54.9)	33 (55.9)	12 (52.2)	…
High (4 or 5 points)	16 (19.5)	8 (13.6)	8 (34.8)	…
BMI (kg/m^2^)	N = 124	N = 87	N = 37	…
	18.5 (16.9–20.4)	18.8 (17.1–20.4)	17.3 (15.4–19.6)	.099
Serum albumin (g/dL)	N = 124	N = 85	N = 39	…
	4.1 (3.8–4.2)	4.1 (3.8–4.3)	3.9 (3.4–4.1)	< .001
C-reactive protein (g/dL)	N = 134	N = 92	N = 42	…
	0.13 (0–0.49)	0.10 (0–0.36)	0.32 (0.06–2.36)	.025
Erythrocyte sedimentation rate (mm/hr)	N = 87	N = 61	N = 26	.127
	22 (13–45)	21 (11–43.5)	29 (15.75–54.25)	…
Positive anti-GPL-core IgA antibody	N = 116	N = 82	N = 34	.235
	102 (87.9)	74 (90.2)	28 (82.3)	…
Never smoker	N = 124	N = 86	N = 38	.415
	92 (74.2)	62 (72.1)	30 (79.0)	…
Previous treatment for NTM-PD	28 (20.7)	14 (15.2)	14 (32.6)	.024
Comorbidities	42 (31.1)	28 (30.4)	14 (32.5)	.804
COPD	4 (3.0)	3 (3.3)	1 (2.3)	…
Bronchial asthma	10 (7.4)	9 (9.78)	1 (2.3)	…
Cured tuberculosis	9 (6.7)	7 (7.6)	2 (4.6)	…
Interstitial lung disease	5 (3.7)	4 (4.4)	1 (2.3)	…
Cured lung cancer	3 (2.2)	3 (3.3)	0	…
Diabetes	13 (9.6)	5 (5.4)	8 (18.6)	…
Autoimmune disease	5 (3.7)	3 (3.3)	2 (4.7)	…
Liver cirrhosis	2 (1.5)	1 (1.1)	1 (2.3)	…
End-stage renal disease	2 (1.5)	1 (1.1)	1 (2.3)	…
Active malignancy	3 (2.2)	2 (2.2)	1 (2.3)	…
Initial treatment regimen				.063
MLs + EMB	8 (6.0)	4 (4.3)	4 (9.3)	…
Daily	7 (5.2)	4 (4.3)	3 (7.0)	…
Three times weekly	1 (0.7)	0	1 (2.3)	…
MLs + EMB + FQs	2 (1.5)	0	2 (4.6)	…
MLs + EMB + RIFs	121 (89.6)	86 (93.5)	35 (81.4)	…
Daily	113 (83.7)	81 (88.0)	32 (74.4)	…
Three times weekly	8 (5.9)	5 (5.4)	3 (7.0)	…
MLs + EMB + RIFs + FQs	1 (0.7)	0	1 (2.3)	…
MLs + EMBs + RIFs + AGs	3 (2.2)	2 (2.2)	1 (2.3)	…
Additional AGs within one year	10 (7.4)	6 (6.5)	4 (9.3)	.566

Data are presented number (%) or median (interquartile range).

Abbreviations: AFB = acid-fast bacilli; AGs = aminoglycosides; BACES = body mass index, age, cavity, bronchiectasis, sex; BE = bronchiectasis; BMI = body mass index; COPD = chronic obstructive pulmonary disease; EMB = ethambutol; FQs = fluoroquinolones; GPL = glycopeptide lipid; MAC-PD = *Mycobacterium avium* complex pulmonary disease; MLs = macrolides; NB = nodular bronchiectatic; NTM-PD = nontuberculous mycobacterial pulmonary disease; PPFE = pleuroparenchymal fibroelastosis; RIFs = rifamycins.

### One-Year Prediction of Treatment Failure

Supplementary Table 1 shows the odds ratios for treatment failure based on the number of lobes with each severity of cavities (any size or > 2 cm in diameter) and bronchiectasis (all, varicose or cystic, and cystic) adjusted by sex, age, and sputum smear positivity. All factors significantly affected treatment failure. We then scored each combination for the extent of the cavities and bronchiectasis as candidates for the predictive model. Table 2 shows the average AUCs for treatment failure in each candidate for the prediction model. Among these candidates, the highest average AUC of 0.801 was found for the sum of the number of lobes with cavities > 2 cm and that with varicose or cystic bronchiectasis. However, the total number of lobes with cavities > 2 cm or with varicose or cystic bronchiectasis showed the second highest average AUC of 0.798, with the receiver operating characteristic curves shown in Supplementary Figure 1. According to the provided average AUCs and the practical use of the calculations, we designated the total number of lobes with cavities (> 2 cm in diameter) or bronchiectasis (varicose or cystic) as the predictive score for treatment failure at 1 year. We named this prediction model the Bronchiectasis and Cavity Distribution (BCD) score. Examples of representative CT findings are presented in Supplementary Figure 2. Calculated weighted kappa for this score was 0.728 (95% confidence interval [CI], .660–.795).

**Table 2.** Average AUCs of Each Combination of Severity of Cavity and Bronchiectasis for Treatment Failure With MAC-PD

Variables	Average AUC
Sum of no. of lobes with cavity and those with BE (maximum 12 points)	
Any cavity and any BE	0.718
Any cavity and varicose or cystic BE	0.771
Any cavity and cystic BE	0.740
Cavity > 2 cm and any BE	0.753
Cavity > 2 cm and varicose or cystic BE	0.801
Cavity > 2 cm and cystic BE	0.754
Total no. of lobes with cavity or BE (maximum 6 points)	
Any cavity or any BE	0.689
Any cavity or varicose or cystic BE	0.775
Any cavity or cystic BE	0.722
Cavity > 2 cm or any BE	0.706
Cavity > 2 cm or varicose or cystic BE	0.798
Cavity > 2 cm or cystic BE	0.761
No. of lobes with single variable (maximum 6 points)	
Any cavity	0.591
Cavity > 2 cm	0.614
Any BE	0.708
Varicose or cystic BE	0.784
Cystic BE	0.733

Average AUCs were calculated using k-fold cross-validation.

Abbreviations: AUC = area under the curve; BE = bronchiectasis; MAC-PD = *Mycobacterium avium* complex pulmonary disease.

### Patient Stratification by BCD Score

Predictive metrics of the BCD score for treatment failure at each cutoff value are shown in Table 3. At the cutoff value ≥ 4 points of the BCD score, specificity was 0.913, positive predictive value was 0.692, and the value of positive likelihood ratio was 4.811. At the cutoff value ≥ 2 points, sensitivity was 0.907, negative predictive value was 0.920, and the value of negative likelihood ratio was 0.186. Based on these characteristics, the patients were stratified into 3 risk classes for treatment failure: low-risk group with a BCD score of 0 or 1 point, intermediate-risk group with 2 or 3 points, and high-risk group with 4 to 6 points (Table 4). The number of patients was well-balanced across the 3 groups, supporting clinical decision-making in initiation of treatment for MAC-PD. The treatment failure rates were 8.0% in the low-risk group, 35.6% in the intermediate-risk group, and 69.2% in the high-risk group. The odds ratios with reference to the low-risk group were 6.355 (95% CI, 2.008–20.118) in the intermediate-risk group and 25.875 (95% CI, 6.925–96.680) in the high-risk group.

**Table 3.** Distribution of Patients and Predictive Metrics for Treatment Failure at Each Cutoff Value of BCD Score

	Cutoff Value						
BCD score	≥ 0	≥ 1	≥ 2	≥ 3	≥ 4	≥ 5	6
Patients (n)	135	117	85	51	26	8	1
Treatment failure (n)	43	41	39	29	18	8	1
Sensitivity	1.000	0.954	0.907	0.674	0.419	0.186	0.023
Specificity	0.000	0.174	0.500	0.761	0.913	1.000	1.000
Positive predictive value	0.319	0.350	0.459	0.569	0.692	1.000	1.000
Negative predictive value	…	0.889	0.920	0.833	0.771	0.724	0.687
Positive likelihood ratio	1.000	1.154	1.814	2.821	4.811	…	…
Negative likelihood ratio	…	0.267	0.186	0.428	0.637	0.814	0.977

Abbreviation: BCD score = Bronchiectasis and Cavity Distribution score.

**Table 4.** Distribution of Patients, Treatment Failure Rates, and Odds Ratios by Risk Classification of BCD Score

BCD Score, Points	0–1	2–3	4–6
Risk Stratification	Low Risk	Intermediate Risk	High Risk
Number of patients (%)	50 (37.0)	59 (43.7)	26 (19.3)
Treatment failure, n (%)	4 (8.0)	21 (35.6)	18 (69.2)
Odds ratio (95% CI)	Reference	6.355 (2.008–20.118)	25.875 (6.925–96.680)

Abbreviations: BCD score = Bronchiectasis and Cavity Distribution score; CI = confidence interval.

### BCD Score Versus BACES Score

We compared predictive accuracy between the BCD score classification and the BACES score classification (13, 17). Among 82 patients for whom the BACES score could be evaluated, the risk classification by BCD score had a higher average AUC (0.759) than the AUC of the BACES score classification (0.649).

## DISCUSSION

In this study, we developed the BCD scoring system to predict treatment failure in patients with MAC-PD using a simple procedure: counting the number of lobes involved with cavities >2 cm in diameter or advanced bronchiectasis of more than the varicose form. This scoring method enabled the 2 raters, both pulmonologists, to perform reasonably consistent evaluations with a weighted kappa of 0.728. This score showed an average AUC of 0.798, whereas predictive accuracy for treatment failure has not been previously reported. These characteristics support the potential use of the score in clinical practice. Moreover, the scoring system stratified the patients into low-, intermediate-, and high-risk classes, which showed gradual elevation of the risk for treatment failure as the risk class increased. Thus, the scoring system and the classification can provide clinicians with guidance on the unresolved clinical question of when to treat patients with MAC-PD.

The study patients' characteristics and treatment success rates were in line with those of previous reports [9, 16, 17, 24]. The characteristics of the treatment failure group indicated the severity of their disease: especially, cavitary lesions and bronchiectasis showed notable differences from those in the culture conversion group. Of note, all study patients received macrolide and ethambutol-containing regimens according to the inclusion and exclusion criteria for treatment. In fact, 95.6% of the study patients received either macrolides + ethambutol + rifamycins, the guideline-recommended regimen [5], or macrolides + ethambutol, which is a potentially standard regimen [25–27]. This treatment uniformity is a strength when evaluating treatment responsiveness of the disease.

The presence of cavities is strongly associated with disease progression, treatment response, and mortality [10, 12, 14–16, 28–31]. In particular, large-size cavities predict disease progression and treatment resistance [15, 31]. Kang and colleagues showed that the presence of cavities > 2 cm significantly influences the failure of a microbiological cure and mortality and suggested that the reasons were a large bacterial load and poor drug penetration in these lesions [15]. Consistent with these findings, the present study showed that the number of lobes involved with cavities > 2 cm in diameter affected treatment failure (adjusted odds ratio = 2.302; 95% CI = 1.375–3.853). Additionally, the number of lobes involved with varicose or cystic bronchiectasis was also associated with treatment response (adjusted odds ratio = 2.688; 95% CI = 1.827–3.955).

Unlike for cavitary lesions, the association of bronchiectasis and disease severity in NTM-PD have not been highlighted previously. However, bronchiectasis is a risk factor for persistent culture positivity [32]. In addition, the extent of bronchiectasis has been suggested to be associated with treatment failure [23] and reported as a predictor of disease progression [11]. Therefore, a clinical association appears to exist between the severity of bronchiectasis and disease activity. Kim and colleagues hypothesized that MAC infection begins in the bronchus, progresses to peribronchial thickening or nodules, develops into cystic bronchiectasis, and manifests as cavitary lesions [33]. This hypothesis suggests that the continuous progression from bronchiectasis to cavities [15] and severe bronchiectasis would serve as an indicator of treatment resistance, analogous to cavities.

Moreover, cavities and bronchiectasis of MAC-PD have been described as irreversible or less responsive changes despite treatment when compared to nodules or bronchiolitis and infiltration on high-resolution CT [34–36]. The BCD score integrates these structural changes of cavities and bronchiectasis, thereby allowing prediction of treatment response in MAC-PD through the evaluation of not only bacterial load and drug penetration but also of irreversibility.

The predictive metrics of the BCD score showed that higher cutoff points accurately predicted treatment failure, whereas lower cutoff points effectively distinguished patients who were successfully treated from those who were not. This graded trend allowed patients to be stratified according to their risk of treatment failure. The risk stratification dividing the patients into 3 classes revealed that risk of treatment failure increases with the extent of destructive lesion.

The stratified risk of treatment failure can guide clinicians in treatment decisions. The patients in the low-risk group, with a BCD score of 0 or 1 point, showed a favorable treatment response, as indicated by an 8.0% treatment failure rate. In this group, watchful waiting with careful follow-up in expectation of spontaneous conversion would be permissible to avoid unnecessary treatment burden. Conversely, if the patients in this group do receive treatment, a highly tolerable treatment regimen such as intermittent treatment or macrolides and ethambutol for patients with rifampin intolerance [25, 37–39] would be preferred. The intermediate-risk group, with a BCD score of 2 or 3 points, exhibited potential treatment resistance: a treatment failure rate of 35.9% and an odds ratio of 6.4 compared to the low-risk group. It is recommended that these patients should be offered treatment that considers their individual backgrounds before the disease progresses. Patients in the high-risk group, with a BCD score of 4 to 6 points, had an apparently high treatment failure rate of 69.2% and an odds ratio of 25.9 in comparison to the low-risk group. The severity of disease in these patients may exceed the point of no return for culture conversion with a guideline-based oral regimen. Therefore, more intensified treatment such as parenteral or inhaled aminoglycosides in addition to the oral regimen, would be required [40–42]. Meanwhile, nonpharmacological interventions to improve patient-reported outcomes, such as rehabilitation and nutritional support [43], are crucial in these patients. These findings emphasize that the appropriate time to initiate treatment for MAC-PD is before the development of structural changes.

In the comparison of predictive accuracy, the BCD score classification showed a higher average AUC than the AUC of the BACES score classification. The BACES score classification is the only scoring system reported to date for predicting culture conversion with treatment in NTM-PD, although it was originally developed to predict mortality in NTM-PD and its application for treatment response prediction was reported in only 1 exploratory study [13, 17]. Despite these limitations, the findings of the present study indicate that the BCD score classification appears to be a reliable predictor of treatment failure. Given its original purpose, the BACES score includes some predictors that are not disease-specific but that affect mortality, such as age and sex [13]. As a result, its ability to directly reflect disease severity itself may be relatively limited. However, as discussed previously, treatment responsiveness would be affected by disease severity itself, including structural changes, more than nonspecific disease factors. For this reason, we suggest that the BCD score, which is based on disease-specific radiographic findings and expression of disease severity, would be better suited to predict treatment response. However, the BCD score should be used as part of a comprehensive evaluation for MAC-PD.

The present study has several limitations. This is a retrospective study conducted at 2 hospitals in the same region, and the number of eligible patients was relatively small. In particular, many patients were excluded mainly because of insufficient sputum culture and cessation of drug treatment. Consequently, there may be selection bias and limited statistical power. Although we performed cross-validation as an internal validation, external validation with large population is still needed to ensure the generalizability and robustness of the study findings. Therefore, a future multicenter prospective study will be needed. Additionally, to maintain simplicity and usability of the scoring system, we selected predictors only from radiographic findings. In clinical practice, treatment indication should be assessed comprehensively along with other clinical factors of the individual patient.

In conclusion, the BCD score, which assesses bronchiectasis and cavitary lesions via CT before treatment, could predict treatment failure and stratify the patients with MAC-PD according to their degree of risk. The simplicity and accuracy of the BCD score provide clinicians with clear guidance to initiate treatment at the optimal timing in patients with MAC-PD. The present study indicated that watchful waiting could be permissible for patients with mild disease. As well, it highlights the importance of initiating treatment before irreversible structural changes develop. Future validation studies with a larger number of participants are warranted.

## Supplementary Material

ofaf565_Supplementary_Data

## Supplementary Data

Supplementary materials are available at *Open Forum Infectious Diseases* online. Consisting of data provided by the authors to benefit the reader, the posted materials are not copyedited and are the sole responsibility of the authors, so questions or comments should be addressed to the corresponding author.
