# Supplementary material for: Developing a Simple Scoring System on CT Findings for Predicting Treatment Failure in Mycobacterium avium Complex Pulmonary Disease: The BCD (Bronchiectasis and Cavity Distribution) Score
Source: Open Forum Infect Dis. 2025 Sep 8;12(9):ofaf565. doi: 10.1093/ofid/ofaf565 (PMC12465108; doi:10.1093/ofid/ofaf565)
Supplement: ofaf565_Supplementary_Data [file ofaf565_supplementary_data.docx]

| **Supplementary Table 1.** Results of multivariate logistic regression models for treatment failure in MAC-PD. | | | |
| --- | --- | --- | --- |
| **Variables** | **Adjusted OR** | **95% CI** | **P value** |
| No. of lobes with cavities | 1.496 | 1.114–2.010 | 0.008 |
| No. of lobes with cavities > 2 cm | 2.302 | 1.375–3.853 | 0.002 |
| No. of lobes with any BE | 1.655 | 1.254–2.185 | < 0.001 |
| No. of lobes with varicose or cystic BE | 2.688 | 1.827–3.955 | < 0.001 |
| No. of lobes with cystic BE | 3.748 | 2.213–6.350 | < 0.001 |
| Each variable was adjusted by sex, age, and sputum smear positivity.  Abbreviations: BE = bronchiectasis; CI = confidence interval; MAC-PD = *Mycobacterium avium* complex-pulmonary disease; OR = odds ratio. | | | |

**Supplementary Figure 1.** ROC curves and AUCs in (A) training and (B) validation data for the BCD score (number of lobes with cavities > 2 cm in diameter or varicose or cystic bronchiectasis). These ROC curves were created using k-fold cross validation (k = 5). AUCs = areas under the curve; BCD = Bronchiectasis and Cavity Distribution; ROC = receiver operating characteristic.


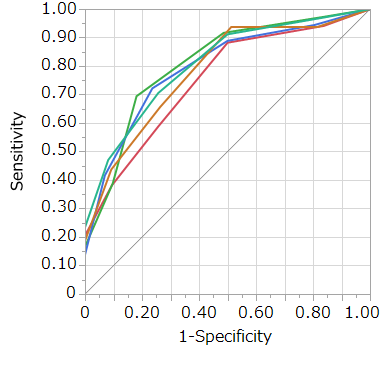

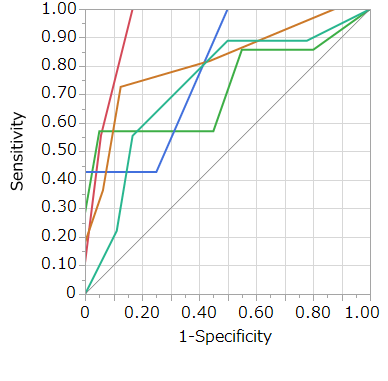

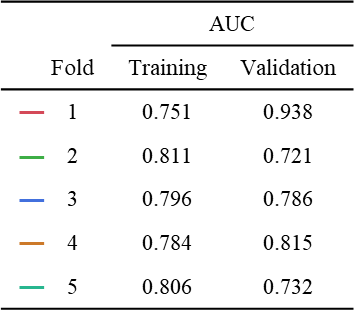


A) Training

B) Validation


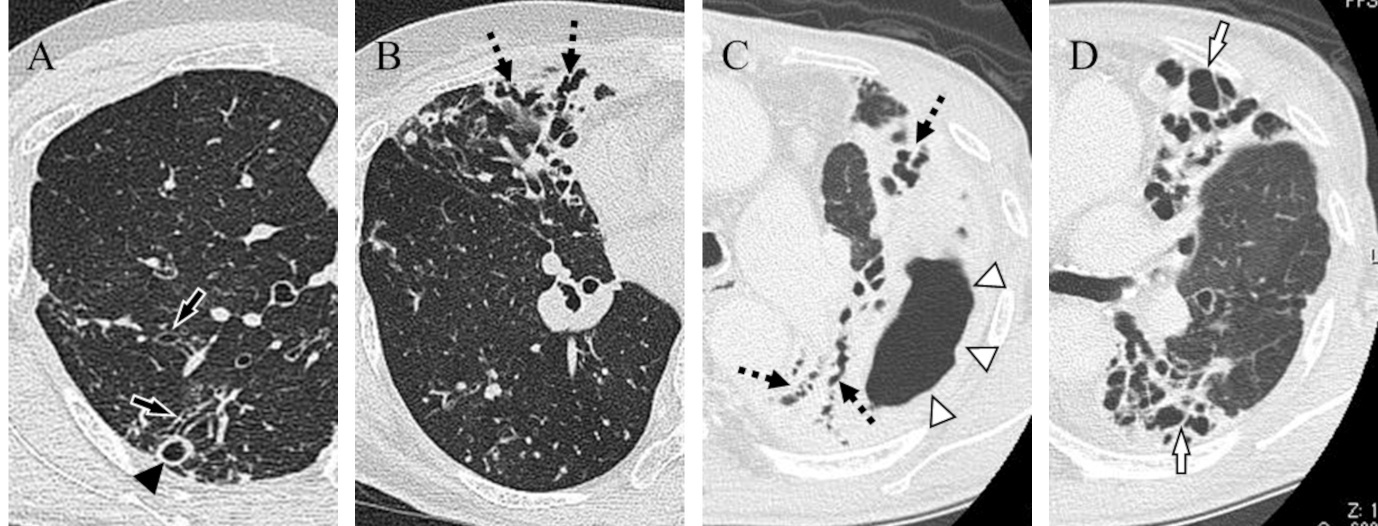


**Supplementary Figure 2.** Pre-treatment CT findings in the assessment of the BCD score. (A, B) A 69-year-old woman with *M. avium* pulmonary disease. She had a BCD score of one point and achieved culture conversion after treatment with azithromycin, ethambutol, and rifampin. (A) The right upper lobe showed cylindrical bronchiectasis (arrows) and a cavity with diameter of 1.0 cm (arrowhead). These findings were not counted for BCD scoring. (B) In contrast, the right middle lobe contains varicose bronchiectasis (dashed arrows), which added one point to the patient’s score. (C, D) A 73-year-old woman with *M. avium* pulmonary disease. Her BCD score was five points, and treatment with clarithromycin, ethambutol, and rifampin did not result in culture conversion at one year. (C) In the left upper segment, a cavity with diameter of 5.4 cm (open arrowheads) and varicose bronchiectasis (dashed arrows) were seen. These findings coexisted in the same segment and were counted as one point. (D) Cystic bronchiectasis (open arrows) was observed both in the lingula and left lower lobe, which added two points to the score. Slice thickness is 1 mm in panels A and B and 1.25 mm in panels C and D. The lung window center is -600 and lung window width is 1500 in all images. BCD = Bronchiectasis and Cavity Distribution; CT = computed tomography.
